# Normal reference ranges of ocular physiology and sonographic biometry of Latvian Hunting dogs

**DOI:** 10.14202/vetworld.2020.807-811

**Published:** 2020-04-28

**Authors:** Līga Kovaļčuka, Gundega Mūrniece

**Affiliations:** Clinical Institute, Faculty of Veterinary Medicine, Latvia University of Life Science and Technologies, Jelgava, Latvia

**Keywords:** anterior chamber depth, axial length, intraocular pressure, Latvian Hunting dogs, lens thickness, *Schirmer tear test*

## Abstract

**Background and Aim:**

The Latvian Hunting dog is the only national dog breed in Latvia and so far, there are no data on the important ophthalmological data pertaining to clinical tests and ocular biometry. The aim of this study was to improve the ocular examination and to document normal reference ranges of the globe axial length (AXL), anterior chamber depth (ACD), lens thickness (LT), intraocular pressure (IOP), and Schirmer tear test (STT).

**Materials and Methods:**

Sixteen Latvian Hunting dogs (32 eyes) of varying age and sex were included in this study. All dogs underwent an ophthalmic examination followed by tear production (STT) and IOP (TonoVet^®^) measurements. Biometric examination using A-mode ultrasonography was done under topical corneal anesthesia using 0.5% proparacaine hydrochloride. The eyes were scanned using A-mode ultrasonography with a 10 MHz transducer by transcorneal approach. The reference values of IOP, STT, AXL, ACD, and LT are expressed as mean ± standard deviation (SD).

**Results:**

Statistical analysis using the SPSS v.23 and Microsoft Excel 2013 showed no statistically significant differences between the right and left eyes for either of the tests. The mean±SD value of STT and IOP values was 20.9±3.2 mm/min and 17.7±3.2 mmHg, respectively. The mean±SD of the AXL, ACD, and LT obtained by A-mode ultrasound was 2.21±0.1 cm, 0.35±0.1cm, and 0.42±0.06cm, respectively.

**Conclusion:**

This study documents the normal reference ranges of important ophthalmological clinical tests (IOP and STT) and ocular biometry (AXL, ACD, and LT) in Latvian Hunting dogs.

## Introduction

**Figure-1:**
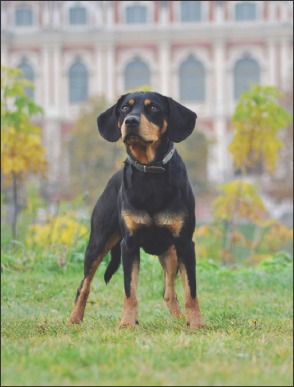
Latvian Hunting dog.

Information about the first and only Latvian national dog breed is not clear. Older sources show that the Latvian Hunting dog was established in the early 17^th^century by Duke Jekab Ketler (1642-1682). The Duke created his own dog breed called “*Kurzemes dzinējsuns*” (Courland Hunting dog). It is presumed that the breed was created by crossing the Luzern Hunting dog, English Hunting dog, and St. Hubert Hunting dog. The Latvian Hunting dog was a popular breed until the First World War, but during the war, the uncontrolled crossing of a number of dog breeds and mixing of other dog blood with the genuine Latvian Hunting dog occurred. Later on, the breed was revived by the careful selection of 40 dogs with the most similar exterior. Only in 1971, the Latvian Hunting dog was established as an official breed (Figure-1). Special features of the particular breed are short, black coat with tan markings, wrinkle-free skin, long, oval ears, forelegs higher than dorsal line, and waist slightly arched [1].

We know that there are differences between dog breeds not only in presumably inherited and congenital diseases but also in physiological rates, such as intraocular pressure (IOP), tear production, and of course anatomical features such as eye globe size and lens size. So far, there are data on Beagle dogs, Samoyeds, and non-specific crossbreed dogs [2-4]. To obtain a full ophthalmological examination, it is important to know the normal ranges of all diagnostic basic tests and anatomical features. As per the authors’ knowledge, no data on the normal reference ranges of the important ophthalmological diagnostic tests and morphometry are available for Latvian Hunting dogs.

In ophthalmology, there are two most important diagnostic tests – Schirmer tear test (STT) and IOP[5]. STT is used to evaluate tear production volume in animals. We know that the pre-ocular tear film is composed of three layers – a mucus layer, an aqueous layer, and a lipid layer. The aqueous layer is produced by the lacrimal and nictitans glands and makes up the bulk of the tear film [6]. During STT measurements, mainly the quantitative aqueous component of the tear film is measured. STT is vital to diagnose a condition characterized by quantitative tear deficiency (keratoconjunctivitis sicca), or conversely, tear over production [5,6]. IOP is a balance between aqueous humor production and outflow [5,7], and it helps to diagnose low-pressure conditions like uveitis and high-pressure conditions like glaucoma [5,8].

Ocular ultrasonography has become one of the essential pre-operative diagnostic and prognostic tools to predict the visual outcome of pets having cataracts, based on the structural integrity of intraocular tissues [9]. Many ultrasonographic studies on the ocular biometry of various dog breeds are available [9-12].

The Latvian Hunting dog is the only national dog breed in Latvia and so far, there are no data on the main ophthalmological clinical tests and morphometric parameters of the eye.

The aim of the study was to improve the ocular examination in Latvian Hunting dogs and to determine the normal range of the following measurements: The globe axial length (AXL), anterior chamber depth (ACD), lens thickness (LT), IOP, and STT-1.

## Materials and Methods

### Ethical approval and informed consent

The study was performed fully respecting the ethical criteria and welfare of the dogs involved. All animals examined were privately owned outpatients. The pre-breeding ophthalmic examination was carried out by the veterinarian/ophthalmologist of the Clinical Institute of the Faculty of Veterinary Medicine at the Latvia University of Life Sciences and Technologies. Informed consent was obtained from the pet owners for the study. The present study did not require specific ethics approval.

### Animals and study design

The study involved 16(5females and 11males) healthy adult Latvian Hunting dogs (32 eyes) between 1 and 13years of age.

To confirm that all dogs were ophthalmologically healthy, complete eye examination was performed. First, basic neurological tests (Menace and Dazzle test, pupillary light reflex) were performed. Eye examination before and after bilateral complete mydriasis caused by tropicamide eye drops (Alcon-Couvreur, Belgium) was performed with direct ophthalmoscopy (Keeler Practitioner, Windsor, UK), monocular ophthalmoscopy with the *PanOptic* ophthalmoscope (Welch Alynn, Romford, UK), and slit-lamp biomicroscopy (Kowa SL15, Nagoya, Aichi, Japan).

Ophthalmological examination of all animals included in this study was conducted by the same person so to ensure uniformity in the results. The age and sex of the animal were recorded.

To perform STT, the standardized sterile strips (Eickemeyer, Tuttlingen, Germany) were used. The tip of the strip was inserted over the lower lateral eyelid margin in the conjunctival fornix for 60 s. After removal of the test strip, the length of the wet part of the strip was immediately measured in millimeters.

All IOP measurements were obtained using a calibrated rebound tonometer (TonoVet^®^, Tiolat Ltd., Finland) on the (d) calibration setting for use in dogs as provided by the manufacturer. Asingle-use probe was positioned perpendicular to the corneal surface approximately 4mm from the central cornea. Each measurement record was the automatically generated average of five successive readings. The use of topical anesthesia is not required when using this tonometer, which benefits the animals since some authors have reported that corneal endothelial and systemic toxicity can occur with the frequent use of topical anesthetics[13,14]. Care was taken to ensure that no compression of the jugular veins or cervical region occurred during measurement.

Biometric examination was done under topical corneal anesthesia by topical application of 0.5% proparacaine hydrochloride (Alcon-Couvreur, Belgium). AXL, ACD, and LT were determined using A-mode ultrasonography with a 10 MHz transducer by transcorneal approach. Local ophthalmic neutral eye gel was applied when the scan was done.

To minimize circadian rhythm influence, all examinations were done at approximately on the same time of day (9.00-11.00 am) as described earlier[15,16].

### Statistical analysis

Statistical analysis of the data was performed using statistical software programs SPSS and Microsoft Excel. The arithmetic mean values (X) and the reference values of IOP, STT, AXL, ACD, and LT were expressed as mean±standard deviation (SD) from each eye separately and both eyes together. Normality was tested using Shapiro–Wilk and Wilcoxon signed-rank test. Anon-paired sample t-test was used to compare the STT, IOP, AXL, ACD, and LT obtained from the right and left eyes, differences between sex and between age groups (1-2years, 2-6years, and older than 6years of age). p<0.05 was considered to be statistically significant.

## Results

In our study, 16 dogs with an average age of 4.7±3.2years and ranging from 1 to 13years were included. There were 11males and 5females. All numeric data obtained for STT test, IOP, AXL, ACD, and LT values in the population used in this study were normally distributed according to the Kolmogorov–Smirnov test.

First, the STT was done in all dogs in both eyes. In our study, the STT showed a mean±SD value of 20.9±3.2mm/min (Table-1), with a median of 21mm/min in both eyes (normal range 14.6–27.3mm/min), showing no significant difference between eyes. No significant differences in the mean values of STT were noticed between the female and male group (p=0.36). A significant difference between the age groups of 1-2years and 2-6years of age was noticed (p=0.02).

**Table-1:** Arithmetic values of STT, IOP AXL, ACD, and LT in Latvian Hunting dog.

M Measurement	Arithmetic mean values±SD			Normal range	Min.	Max.
IOP (mmHg)	17.7±3.2			11.3-24.1	11.0	24.0
	1-2 y.o.	2-6 y.o.	>6 y.o.			
	18.5±2.4	17.0±3.3	19.4±0.9			
STT (mm/min)	20.9±3.2			14.6-27.3	16.0	30.0
	1-2 y.o.	2-6 y.o.	>6 y.o.			
	18.5±2.1	22.5±2.6	20.8±3.5			
AXL (cm)	2.21±0.08			2.05-2.37	2.1	2.3
	1-2 y.o.	2-6 y.o.	>6 y.o.			
	2.07±0.1	2.16±0.05	2.17±0.04			
ACD (cm)	0.35±0.1			0.13-0.6	0.22	0.53
	1-2 y.o.	2-6 y.o.	>6 y.o.			
	0.34±0.1	0.31±0.05	0.5±0.04			
LT (cm)	0.42±0.06			0.29-0.55	0.3	0.52
	1-2 y.o.	2-6 y.o.	>6 y.o.			
	0.30±0.01	0.40±0.01	0.47±0.07			

SD: Standard deviation, IOP=Intraocular pressure, y.o.=years old, STT=*Schirmer tear* test, AXL=Globe axial length, ACD=Anterior chamber depth, LT=Lens thickness

The IOP value in this study showed a mean±SD of 17.7±3.2mmHg in both eyes (normal range 11.3-24.1mmHg), as presented in Table-1, with a median of 17.5mmHg and showing no significant differences between eyes. There were no significant between the right and left eye (p=0.2) gender differences in IOP (p=0.47), or between the age groups of 1-2years and 2-6years (p=0.26), although a significant difference between the age groups of 2 and 6years and dogs older than 6years of age was noticed (p=0.009): IOP was significantly higher in the oldest age group compared to the middle-age group.

Eye anatomical measurements AXL, ACD, and LT were obtained using A-mode ultrasound. In our study, mean AXL size was 2.21±0.08cm, varying from 2.1 to 2.3cm, with normal range 2.05-2.37cm (Table-1). Average ACD was 0.35±0.1cm in both eyes, varying from 0.22 to 0.53cm, with normal range 0.13-0.6cm (Table-1). The most important structure for intraocular surgery is LT, in our study, the average size was 0.42±0.06cm, varying from 0.3 to 0.52cm, with normal range 0.29-0.55cm (Table-1). In all measurements, no significant differences were noticed between different animal sexes or between age groups (p>0.05).

## Discussion

To the authors’ knowledge, this is the first study made in this particular dog breed. It is important to establish normal reference values of various diagnostic tests in healthy dogs of various dog breeds to avoid diagnostic misinterpretations during the ophthalmic examination.

First, the SST strips were placed in the ventral conjunctival sack without difficulty and all dogs tolerated the test well. The STT has long been the clinical standard for evaluating aqueous tear production[17,18] and general reference values are known in different animal species such as dogs, cats, rabbits, and horses [19-24], and presumably, these values may differ among various dog breeds [23-25].

In our study on Latvian Hunting dogs, STT showed a mean of 20.9±3.2mm/min in both eyes (range 14.6-27.3mm/min) which was similar to in comparison with reported values in other dog breeds where STT values ranged from 7 to 35mm/min and the overall average STT value of 400 measurements was 20.4±2.89mm/min [25]. In another study, in 16 mixed-breed dogs, mean STT measured in the ventral conjunctival fornice was 23.56±3.98mm/min, which was higher values than measured in the dorsal fornice and comparable to our study. [26]. Results presented in our study were obtained by placing the strip in ventral conjunctival fornix, similar to other studies[26,27], the mean STT value was 20.9±3.2 (range= 14.6-27.3mm/min).

There are not so many studies showing reference values in specific breeds, but the mean STT value in Turkish Hunting dogs was 17.1±3.7mm/min[23], Beagles 20±2.5mm/min, Labrador Retriever 22.9±4.1mm/min, English Springer Spaniel 20.7±3.2mm/min, Golden Retriever 21.8±3.7mm/min, and Shetland sheepdog 15.8±1.8mm/min [2], so overall the mean STT is similar to our result of 20.9±3.2mm/min.

Variations in STT values with age, gender, and weight have been studied previously, with some studies demonstrating no statistically significant differences [23] and others showing a significant effect on STT measurements [28,29]. Our study demonstrates no statistically significant difference between STT values in dogs of different gender and between the right and left eyes (p>0.05), but significant difference between the age groups of 1-2years and 2-6years of age was noticed (p=0.02).

Overall, we need to mention that our results were in the normal reference range of STT values, but our study gives a more narrow and precise normal range for this specific breed and that should be taken into account when performing the ophthalmological examination in Latvian Hunting dogs.

The current study was also undertaken to establish reference values of IOP in clinically normal Latvian Hunting dogs with the rebound tonometer. In Latvian Hunting dogs, we calculated the normal range of IOP as 11.3-24.1mmHg (mean±SD of 17.7±3.2mmHg in both eyes). These values are slightly higher than in a previous study of mixed dog breeds, where IOP ranged from 12.8±1.8mmHg to 17.7±3.1mmHg [30] and similar to other author reported values – from 16.7±4.0mmHg to 18.7±5.5mmHg [7,31].

As mentioned, we measured IOP at approximately the same time of a day (9-11 a.m.) to avoid circadian rhythm influence, as some studies show that it can have an effect on IOP measurements, for example, in the evening, IOP may decrease [32]. Concerning IOP normal range in a specific dog breed, a study of Turkish Hunting dogs presented that IOP was 17.63±3.34mmHg using the TonoVet^®^ and there was no effect of sex, age, or eye side (right vs. left)[3]. These results are similar to our research, where no significant differences were noticed between different animal sexes. On the contrary, there was a significant difference between the age groups of 2-6 and older than 6years.

These days there are many methods such as MRI, CT, and B-mode ultrasound to measure the size of eye anatomical structures and dimensions (AXL, ACD, and LT). In this study, A-mode ultrasound was used for measuring ocular biometry. Ultrasound is a non-invasive, non-painful procedure where no animal sedation is needed and also a more economical method of examination. A-mode, by comparison to B-mode, is a two-dimensional, simpler method. These measurements are fundamentally important to characterize the eye anatomy in Latvian Hunting dogs and more importantly, these data are essential clinically before any intraocular surgery, especially cataract surgery. In this study, mean AXL size was 2.21±0.1cm, varying from 2.05 to 2.37cm. In the published literature, where measurements were made using CT, AXL in normal dogs’ eyes was 2.09cm [33] that was a little shorter than in Latvian Hunting dogs.

Shorter AXL has been reported in crossbreed dogs that weighed 5-12kg – 1.75±1.0 [10], we need to mention that Latvian Hunting dogs in our research, weigh was 15-16kg. In a study by Cottrill *etal*.[11], AXL was compared in dolichocephalic dogs (2.12±1.3cm) and mesocephalic dogs (1.99±1.2). The Latvian Hunting dog would be more like mesocephalic dog, but AXL in our research was longer than that reported in dolichocephalic dogs [11].

Values of ACD are varying in literature, we found an average ACD of 0.35±0.1cm in both eyes, similarly, in mixed dogs, ACD was 0.4cm [33], 3.8±0.1cm [34], but Cottrill *et al*. [11] found a shallower anterior chamber in dogs (0.15-0.20cm). Miller and Murphy showed that the anterior chamber readings by ultrasound could have a 0.14cm measurement error because of corneal flattening using an ultrasound probe [34]. In our research, we used an A-mode probe that is smaller than a B-mode probe and exerts less pressure on the cornea but also should be placed on the surface of the central cornea, with no pressure made to the cornea. In addition, we need to note that there were significant differences between A-and B-modes ultrasonographic readings when different structures of the eye were evaluated [12].

A most important structure in intraocular surgery is LT, in our study, the average size was 0.42±0.1cm, which is much thinner than in the previous studies; in mixed-breed dogs, LT was 0.7cm, in English Cocker Spaniels, it was 0.71cm [35], and in a study by Williams in 50 normal dogs, LT was 0.6±0.1cm obtained by B-mode ultrasonography dogs [9].

## Conclusion

This study is the first study providing reference ranges for STT, IOP, AXL, ACD, and LT in normal eyes of Latvian Hunting dogs.

## Authors’ Contributions

LK conceptualized the aim of the study, designed, planned, supervised the analysis, and corrected the manuscript. LK and GM conceived the work, performed all animal examinations and tests, analyzed the data, prepared the graphs, figures, and tables, and drafted the manuscript. Both authors have read and approved the final manuscript.
